# Predictive equations for evaluation for resting energy expenditure in Brazilian patients with type 2 diabetes: what can we use?

**DOI:** 10.1186/s40795-020-00384-1

**Published:** 2020-09-30

**Authors:** Thaiciane Grassi, Francesco Pinto Boeno, Mauren Minuzzo de Freitas, Tatiana Pedroso de Paula, Luciana Vercoza Viana, Alvaro Reischak de Oliveira, Thais Steemburgo

**Affiliations:** 1 Postgraduate Program in Food, Nutrition, and Health, Universidade Federal do Rio Grande do Sul (UFRGS), Ramiro Barcelos Street 2400, 2nd Floor, Porto Alegre, RS 90035-003 Brazil; 2 School of Physical Education, UFRGS, Porto Alegre, RS Brazil; 3 Endocrine Division, Hospital de Clínicas de Porto Alegre, Porto Alegre, RS Brazil

**Keywords:** Indirect calorimetry, Type 2 diabetes, Resting energy expenditure, Energy metabolism

## Abstract

**Background:**

Evaluation of the resting energy expenditure (REE) is essential to ensure an appropriate dietary prescription for patients with type 2 diabetes. The aim of this record was to evaluate the accuracy of predictive equations for REE estimation in patients with type 2 diabetes, considering indirect calorimetry (IC) as the reference method.

**Methods:**

A cross-sectional study was performed in outpatients with type 2 diabetes. Clinical, body composition by electrical bioimpedance and laboratory variables were evaluated. The REE was measured by IC (QUARK RMR, Cosmed, Rome, Italy) and estimated by eleven predictive equations. Data were analyzed using Bland–Altman plots, paired *t*-tests, and Pearson’s correlation coefficients.

**Results:**

Sixty-two patients were evaluated [50% female; mean age 63.1 ± 5.2 years; diabetes duration of 11 (1–36) years, and mean A1C of 7.6 ± 1.2%]. There was a wide variation in the accuracy of REE values predicted by equations when compared to IC REE measurement. In all patients, Ikeda and Mifflin St-Jeor equations were that most underestimated REE. And, the equations that overestimated the REE were proposed by Dietary Reference Intakes and Huang. The most accurate equations were FAO/WHO/UNO in women (− 1.8% difference) and Oxford in men (− 1.3% difference).

**Conclusion:**

In patients with type 2 diabetes, in the absence of IC, FAO/WHO/UNO and Oxford equations provide the best REE prediction in comparison to measured REE for women and men, respectively.

## Background

Type 2 diabetes is the most common form of diabetes and is associated with obesity in about 80% of cases [1, 2]. The main treatment strategy for obese people with type 2 diabetes is improved glycemic control by weight loss [2]. Therefore, an accurate assessment of resting energy expenditure (REE) is essential for an adequate dietary prescription to reduce body weight [3]. The most precise procedure for measuring REE is indirect calorimetry (IC), which is considered the reference method [3]. However technical difficulties hinder its use and predictive equations are largely used instead [4–14].

Several factors have been shown to influence REE, such as sex, ethnicity, age, physical activity, genetic factors, body composition, caloric intake, and the presence of diabetes or obesity [11]. Research conducted in different populations [15–17] and ethinicities [18–25] have evaluated REE using predictive equations. Studies considering sex have shown that REE is lower in women than in men [26–28]; one such study found that REE measured by IC was 23% higher in men [26]. These data contributed to a follow-up study conducted in obese men and women, which also demonstrated a significant difference (REE higher in men by approximately 335 kcal/day) [28]. In fact, the differences between the male and female gender in BMR are primarily attributed to differences in body size and composition [26–28].

In addition, the presence of diabetes is also associated with REE. Previous studies demonstrated that patients with diabetes and poor glycemic control had higher REE [9, 24, 25]. Data on the use of REE predictive equations in patients with type 2 diabetes have been described elsewhere [9, 10, 14, 20, 21, 23–25, 29–34]; however, data on Brazilian diabetic patients are still scarce [33, 34]. A cross-sectional study of obese Brazilian women with type 2 diabetes showed that some predictive equations underestimated REE by approximately − 2.6%, while others overestimated it by 10.6%, when compared with IC measurement [33]. A recent study by our research group conducted on Brazilian patients with type 2 diabetes of both sexes, we found a wide variation in REE values evaluated by predictive equations. The FAO / WHO / UNO equation showed the best precision when compared with the measured REE, but still underestimated it by − 5.6% compared to CI, a difference of 100 kcal / day. In addition, sex was correlated with the REE measured by IC, and it is important to carry out another study to assess the differences between men and women [34].

Considering that sex is an important variable in REE evaluation; that data in Brazilian patients with type 2 diabetes are insufficient; and that poor glycemic control has been associated with an increase in REE, evaluating the performance of predictive equations for REE in this population is essential to ensure that adequate dietary interventions are being prescribed for diabetic patients. Within this context, the aim of the present study was to evaluate the accuracy of the main predictive equations used in clinical practice for the calculation of REE in a sample of Brazilian patients with type 2 diabetes, according to sex, considering IC as the reference method.

## Methods

### Study subjects

This study was designed and reported according to the Strengthening the Reporting of Observational studies in Epidemiology (STROBE) Statement providing all sections suggested to cross-sectional studies.

### Study setting

This cross-sectional study was conducted in outpatients with type 2 diabetes at the Endocrinology Division, Hospital de Clínicas de Porto Alegre, Brazil.

### Participants

Patients with type 2 diabetes who had not received any dietary counselling by a registered dietitian during the previous 6 months were eligible. Other inclusion criteria were age < 70 years, serum creatinine < 2 mg/dL, normal thyroid function tests, and absence of severe liver disease, decompensated heart failure, or any acute disease. Patients underwent clinical, laboratory, and nutritional evaluation. All medications in use were maintained during the study. Diabetes was defined as onset of hyperglycemia over 30 years of age, with no previous episode of ketoacidosis or documented ketonuria and treatment with insulin only after 5 years of diagnosis. All procedures involving patients were approved by Ethics Committee and written informed consent was obtained from all patients.

### Eligibility

**Fig. 1:**
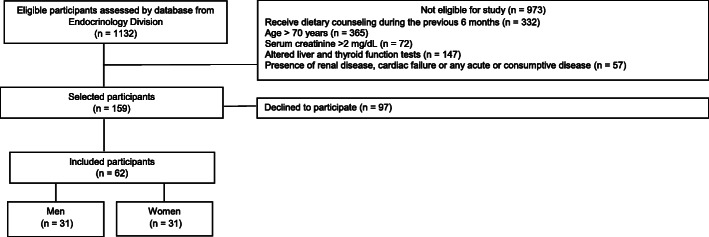
Flowchart of patient selection

The patients’ eligibility was verified from the Endocrinology Division database, where all patients meeting the eligibility criteria were selected. Of 1132 patients medical records screened, 973 were automatically excluded due to receive dietary counseling from a registered dietitian during the previous 6 months (*n* = 332), age > 70 years old (*n* = 365), serum creatinine > 2 mg/dl (*n* = 72), altered liver and thyroid function tests (*n* = 147), and presence of renal disease, cardiac failure, or any acute or consumptive disease (*n* = 57). Of 159 eligible screened patients, 97 were excluded because declined to participate. Final analyses were performed per protocol, and we included 62 patients patients with type 2 diabetes (31 men and 31 women). Of these 62 patients, data from 21 patients were used according to a study previously published by our research group [34]. The flow diagram of patient selection is shown in Fig. 1.

### Data sources / measurements

#### Clinical evaluation

The body weight and height of patients (without shoes and coats) were obtained using a calibrated and anthropometric scale (Filizola®). Measurements were recorded to the nearest 100 g for weight and to the nearest 0.1 cm for height. Body mass index (BMI) was calculated as weight in kilograms divided by the square of the height in meters. The body composition was performed by means of the electrical bioimpedance (InBody® 230, Seoul, South Korea) for the determination of fat mass (FM) (kg) and fat-free mass (FFM) (kg).

Usual physical activity was objectively measured by step counting with a pedometer (HJ-321, Omron® Health Care Co.) and was classified into five levels: sedentary (< 5.000 step/day), low active (5.000–7.499 step/day), somewhat active (7.500–9.999 step/day), active (> or = 10.000–12.499 step/day) and highly active (> or = 12.500 step/day) [35]. Participants wore pedometer for 7 days, attached to the waistband of their clothing during waking hours, except when bathing or swimming. Participants were encouraged not to alter their usual physical habits during protocol.

Blood pressure was measured twice to the nearest 2 mmHg, after a 10 min rest, using an Omron HEM-705CP digital sphygmomanometer (Omron Healthcare, Inc., Bamockburn, IL, USA). Hypertension was defined as blood pressure ≥ 140/90 mmHg measured on two occasions, history of hypertension or the use of antihypertensive drugs.

#### Laboratory evaluation

Blood samples were obtained after a 12-h fast. Plasma glucose level was determined by the glucose-peroxidase enzymatic colorimetric method (Bio Diagnóstica), HbA1C by high-performance liquid chromatography (Merck-Hitachi L-9100, Merck Diagnostica, Darmstadt, Germany; reference range, 4.8–6.0%), total cholesterol and triglycerides by enzymatic colorimetric methods (Merck; Boehringer Mannheim, Buenos Aires, Argentina), and high-density lipoprotein (HDL) by a homogeneous direct method (AutoAnalyzer, ADVIA 1650). Low-density lipoprotein (LDL) cholesterol was calculated using the Friedewald formula (LDL cholesterol = total cholesterol – HDL cholesterol – triglycerides/5).

#### Resting energy expenditure measurement

The measurement of REE was performed by IC. The IC protocol consisted of 10 min of rest on a gurney in dorsal decubitus, followed by 30 min of collection of exhaled gases using a mask and a coupled collection device. An open-circuit calorimeter (QUARK RMR, Cosmed, Rome, Italy) was used to determine VO_2_ (oxygen consumption) and VCO_2_ (carbon dioxide production). The first 10 min of gas collection were excluded from the analysis; thus, VO_2_ and VCO_2_ (l/min) obtained during the final 20 min of each collection (mean value of the period) were used for the calculation of REE. The equation proposed by Weir was used to obtain values in kcal/min, which does not require the use of protein metabolism by incorporating a correction factor [36]. Subjects were asked to refrain from all moderate- or high-intensity physical activity during the 24 h preceding the test, and not to consume alcohol or caffeine. Smokers were instructed not to consume any tobacco products for at least 12 h before the day of REE measurement. Additionally, the subjects were instructed to fast for 12 h prior to the test (water freely allowed) and to have a good night’s sleep (at least 8 h). Finally, all subjects either drove or were driven to the test site to avoid any energy expenditure before determination of REE. All tests were performed between 06:30 and 08:00, in a temperature-controlled (23 °C) and sound-controlled room, under low luminosity. All medications in use were maintained during the study period and patients received their usual medication after the IC.

#### Selection of equations for estimating resting energy expenditure

The REE was estimated by eleven predictive equations, which were selected after a search of previous publications on the them: Harris-Benedict [4], Bernstein [5], Schofield [6], FAO/WHO/UNO [7], Mifflin-St. Jeor [8], Gougeon [9], Huang [10], Martin [11], Dietary Reference Intakes (DRIs) proposed by Institute Of Medicine [12], Oxford [13] and Ikeda [14]. To be included, the equations had to have been developed for adult men and women and should be based on body weight, height, age, sex, and/or FM. Equations derived only for specific ethnic groups or for individuals with BMI ≥40 kg/m^2^ were not included (**Supplement** **1**).

### Sample size

Sample size calculation was based on a study wherein the variability of REE in relation to glycemic control, weight, age, and sex—particularly in male patients—demonstrated a multiple correlation coefficient of 0.9 [24]. Considering a study power of 80%, alpha error of 5, and 20% attrition rate, 62 patients would be required.

### Statistical analysis

Results are expressed as means and standard deviations or medians and interquartile ranges. The Shapiro-Wilk normality test was used to determine the distribution of the variables.

The bias was calculated by subtracting the measured REE from the estimated REE. The means of estimated REE and measured REE were compared by a paired Student’s t-test. Agreement between estimated and measured REE was examined graphically by plotting the differences between the predicted and the measured REE against their mean values, with 95% limits of agreement (mean difference ± 1.96 standard deviation) [37]. Pearson’s correlation coefficients were used to assess the correlation between the estimated and measured REE and to assess the correlation between the dependent variable between dependent and independent variables. Data were analyzed using SPSS version 23.0, while Bland–Altman plot values were analyzed in R version 3.3.3 (R Project for Statistical Computing, Vienna, Austria). For all tests, a *p* value < 0.05 was considered statistically significant.

## Results

A total of 62 patients with type 2 diabetes (31 men and 31 women) were evaluated in this record. A flow diagram of patient selection is shown in Fig. 1. Most of the patients were white (80.6%) and mean age was 63.1 ± 5.2 years old, median disease duration was 11 [1–35, 38] years and mean BMI, 30.1 ± 4.0 kg/m^2^. Men had greater body mass (89.9 ± 13.8 vs. 74.2 ± 11; *p* < 0.001) and FFM (38.6 ± 12.1 vs. 31.7 ± 10.7; *p* = 0.009) when compared to women. Regarding physical activity, the median number of steps/weeks was 5522 (1496–18,097), thus classifying the majority of participants as less active. All participants (100%) had hypertension. Most had a lipid profile within normal limits; however, fasting blood glucose and A1c levels were abnormal, as expected in a sample of patients with diabetes. All were on oral antihyperglycemic agents (100%) and antihypertensive agents (100%), while 67.7% (*n* = 42) also took lipid-lowering agents. The profile of the sample is described in Table 1.

**Table 1:** Sample profile

Variable	Overall (***n*** = 62)	Men (***n*** = 31)	Women (***n*** = 31)	***p*** value
Age (years)	63.1 ± 5.2	63.5 ± 5.5	62.6 ± 4.9	0.473^a^
Duration of diabetes (years) Ethnicity (white)	11 (1–36) 50 (80.6%)	12 (1–36) 28 (90.3%)	10 (2–30) 22 (71%)	0.493^b^ 0.307^a^
Weight (kg)	82.1 ± 14.8	89.9 ± 13.8	74.2 ± 11.2	**< 0.001**^a^
Height (cm)	164.8 ± 10.3	172.4 ± 7.6	157.2 ± 6.2	**< 0.001**^a^
BMI (kg/m^2^)	30.1 ± 4.0	30.3 ± 3.8	30.0 ± 4.2	0.736^a^
Fat-free mass (kg)	35.2 ± 11.8	38.6 ± 12.1	31.7 ± 10.7	**0.009**^a^
Fat mass (Kg)	29.1 ± 8.8	27.9 ± 9.3	30.3 ± 8.3	0.278^a^
Physical activity (steps/week)	5522 (1496–18,097)	5190 (1496–18,097)	6011(1941–14,316)	0.288^a^
Hypertension	62 (100%)	31 (100%)	31 (100%)	–
Fasting plasma glucose (mg/dL)	153.3 ± 46.2	162.9 ± 45.5	143.8 ± 45.6	0.105^a^
A1C (%)	7.6 (5.2–12.0)	7.9 (5.9–12.0)	7.2 (5.2–9.2)	0.126^b^
Total cholesterol (mg/dL)	162.5 ± 40.3	158.0 ± 44.4	171.1 ± 33.7	0.197^b^
HDL cholesterol (mg/dL)	44.7 ± 13.8	39.8 ± 8.7	52.7 ± 13.8	**< 0.001**^**b**^
Triglycerides (mg/dL)	172 (49–681)	183 (49–681)	157 (68–342)	0.789^b^
Medications				
Oral antihyperglycemic agents	62 (100%)	31 (100%)	31 (100%)	–
Antihypertensive agents	62 (100%)	31 (100%)	31 (100%)	–
Hypolipidemic agents	42 (67.7%)	22 (71%)	20 (64.5%)	0.587^c^

*BMI* Body mass index; *A1C* Glycated hemoglobin; *HDL* High-density lipoprotein

Data presented as median (interquartile range), n (%), or mean ± standard deviation

^a^ Student’s *t*-test; ^b^ Mann–Whitney *U* test; ^c^ Chi-square test

— Chi-square test impossible because 100% of the sample is hypertensive, on hypoglycemic agents, and on antihypertensive agents

Table 2 shows the mean and standard deviation of REE as measured by IC and estimated by the predictive equations, bias (percent deviation), and 95% limits of agreement. All variables were normally distributed according to the Shapiro-Wilk test (data not shown). The mean REE measured by IC in men and women was 1815.7 ± 262.3 kcal/day and 1473.4 ± 258.5 kcal/day respectively (*p* < 0.001). In all patients, only the Bernstein equation showed no statistically significant difference in relation to REE measured by IC. When stratified by sex, in men, the Harris-Benedict, FAO/WHO/UNO, and Oxford equations did not yield results significantly different from REE measured directly by IC. However, the Oxford equation presented a smaller value of bias, around − 1.3%, and for clinical practice this corresponds to 54 kcal/day. In women, only the FAO/WHO/UNO equation did not differ significantly from REE as measured by IC.

**Table 2:** Evaluation of measured and estimated REE in patients with type 2 diabetes

	All (***n*** = 62)				Men (***n*** = 31)				Women (***n*** = 31)			
	Mean	SD	95% limits of agreement^1^	*p* value*	Mean	SD	95% limits of agreement^1^	*p* value*	Mean	SD	95% limits of agreement^1^	*p* value*
Measured REE by IC (kcal/day)	1644.6	310.6			1815.7	262.3			1473.4	258.5		
Estimated REE (kcal/day)												
**Harris-Benedict** [4]	1546.9	262.5		**0.001**	1734.4	231.8		0.065	1359.3	117.1		**0.004**
Bias^2^ (kcal/day)	−97.7		(− 153.4;-41.94)		−81.3		(− 167.8; 5.3)		−114.1		(− 189.0; −39.2)	
Percent deviation^3^	−3.5				−1.9				−3.1			
**Bernstei n**[5]	1660.1	498.9		0.799	2045.1	438.2		**0.031**	1275.0	88.1		**< 0.001**
Bias^2^ (kcal/day)	15.5		(−105.3; 136.3)		229.4		(22.1; 436.6)		−198.4		(− 277.5;-119.3)	
Percent deviation^3^	0.2				2.2				−5.1			
**Schofield** [6]	1478.5	296.7		**< 0.001**	1700.1	225.9		**0.011**	1256.8	162.6		**< 0.001**
Bias^2^ (kcal/day)	− 166.1		(−223.6;-108.5)		−115.6		(− 203.2; −27.9)		− 216.6		(− 291.7; 141.5)	
Percent deviation^3^	−5.7				−2.6				−5.8			
**FAO/WHO/UNO** [7]	1603.2	257.3		**0.016**	1745.6	227.7		0.108	1407.8	119.3		0.074
Bias^2^ (kcal/day)	−41.4		(− 122.6; −13.0)		−70.1		(− 156.6; 16.3)		−65.6		(−137.9; 6.7)	
Percent deviation^3^	−2.4				−1.6				−1.8			
**Mifflin–St.Jeor** [8]	1454.9	264.4		**< 0.001**	1663.5	181.7		**0.001**	1246.3	138.7		**< 0.001**
Bias^2^ (kcal/day)	−189.7		(− 243.7;-135.6)		−152.2		(−235.3; −69.0)		−227.1		(− 298.5;155.7)	
Percent deviation^3^	−7.0				−3.7				−6.4			
**Gougeon et al.** [9]	1547.1	248.0		**0.002**	1715.4	196.5		**0.028**	1378.8	167.2		**0.028**
Bias^2^ (kcal/day)	−97.5		(−156.5; − 38.3)		−100.3		(− 188.8; − 11.7)		−94.6		(− 178.1; −	
Percent deviation^3^	−3.2				−2.3				−2.3		11.1)	
**Huang et al.** [10]	2052.6	369.8		**< 0.001**	2248.5	345.0		**< 0.001**	1856.7	282.4		**< 0.001**
Bias^2^ (kcal/day)	408		(337.6; 478.4)		432.8		(324.6;		383.3		(287.5; 478.9)	
Percent deviation^3^	11.5				8.1		541.0)		8.1			
**Martin et al.** [11]	1330.6	236.7		**< 0.001**	1428.2	228.0		**< 0.001**	1233	205.6		**< 0.001**
Bias^2^ (kcal/day)	− 314		(−382.6; − 245.2)		−387.5		(− 491.9; − 283.0)		−240.4		(−327.4;-153.3)	
Percent deviation^3^	−9.1				−7.5				−5.6			
**Dietary Reference Intakes** [12]	2086.8	372.5		**< 0.001**	2395.4	228.0		**< 0.001**	1778.1	186.7		**< 0.001**
Bias^2^ (kcal/day)	442.2		(375.9; 508.4)		579.7		(495.3; 664.0)		304.7		(− 225.6; 383.7)	
Percent deviation^3^	13.3				14.0				7.8			
**Oxford** [13]	1556.1	271.6		**0.002**	1761.7	218.9		0.201	1350.4	121.3		**0.002**
Bias^2^ (kcal/day)	−88.5		(− 143.3; −33.6)		−54		(− 138.4; 30.3)		− 123		(− 195.8;-50.1)	
Percent deviation^3^	−3.2				−1.3				−3.4			
**Ikeda et al.** [14]	1381.7	151.8		**< 0.001**	1458.6	147.6		**< 0.001**	1304.8	113.4		**< 0.001**
Bias^2^ (kcal/day)	−262.9		(−321.9; −203.7)		− 357.1		(−436.1; − 278.0)		− 168.6		(− 246.7;-90.5)	
Percent deviation^3^	−8.8				−9.2				−4.4			

*REE* Resting energy expenditure; *IC* Indirect calorimetry; *SD* Standard deviation

^*^ Paired Student’s *t-*test to compare estimated and measured REE

^1^ (mean difference ± 1.96 SD of the difference)

^2^ (estimated − measured) (kcal in 24 h)

^3^ (difference/measured) × 100 (%)

According to percent variation, the predictive equations that most underestimated REE as compared to IC was that of Ikeda in men (− 9.2%) and Mifflin St-Jeor in women (− 6.4%). The equation proposed by Bernstein underestimated the measured REE in men (− 5.1%) and overestimated it in women (2.2%). The equations that presented the best accuracy were Oxford for men (− 1.3%) and FAO/WHO/UNO for women (− 1.8%), with a precision of 54 kcal and 65.6 kcal/day, respectively.

**Fig. 2:**
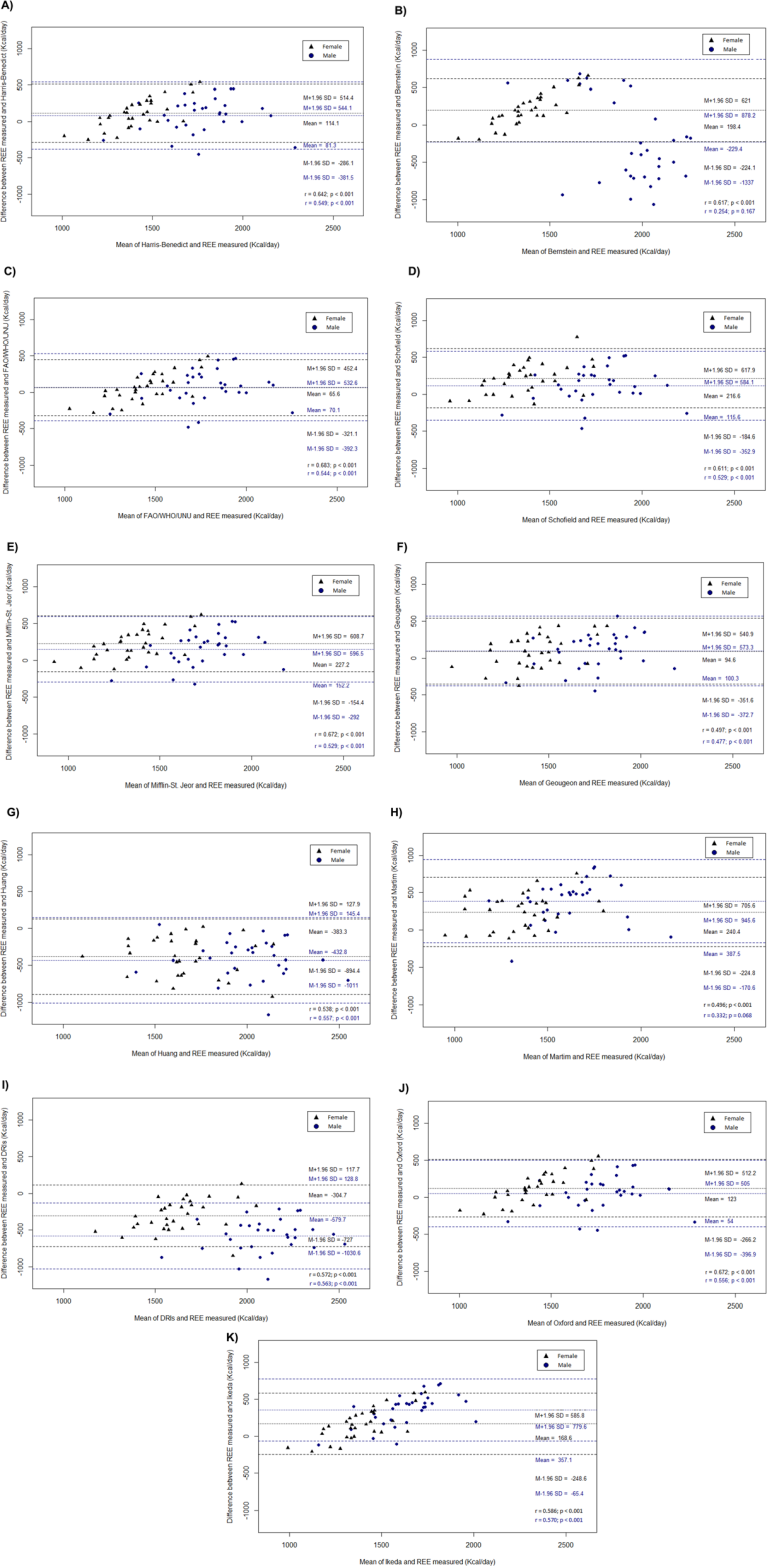
Bland–Altman plots comparing indirect calorimetry (IC) and the following predictive equations for resting energy expenditure (REE) in patients with type 2 diabetes: **a**) Harris-Benedict [4]; **b**) Bernstein [5]; **c**) FAO/WHO/UNO [6]; **d**) Schofield [7]; **e**) Mifflin–St.Jeor [8]; **f**) Gougeon et al [9]; **g**) Huang et al [10]; **h**) Martin et al [11]; **i**) DRIs, Dietary References Intakes [12]; **j**) Oxford [13]; and **k**) Ikeda et al [14]

Figure 2 shows the differences in mean REE measured by IC and that estimated by the predictive equations. The Bland–Altman plots suggest poor correlation between measured and estimated REE, with broad concordance limits. The lower and upper limits are always higher in men, indicating that REE variation is greater in this group. Positive, significant correlations were observed in both sexes between IC-measured REE and with most of the predictive equations. In men, only Bernstein’s proposed equation showed no correlation with IC-measured REE measured by IC. Correlation analysis also showed a significant association (*p* < 0.001) between dependent and independent variables in both sexes. In women, REE correlated positively with weight (*r* = 0.538), height (*r* = 0.516), and FFM (*r* = 0.492). In men, REE correlated with weight (*r* = 0.557), BMI (*r* = 0.545), and FM (*r* = 0.482). We did not observe significant correlations between REE and glycemic control in this group of patients.

## Discussion

Few studies have compared REE values measured by IC versus those estimated by predictive equations in Brazilian patients with type 2 diabetes [33, 34]. The REE values predicted by the Oxford and FAO/WHO/UNO equations, in men and women respectively, were those closest to IC-measured REE in our sample. Our results are consistent with those of a previous study conducted in Brazilians with type 2 diabetes, in which the FAO/WHO/UNO equation had the best performance for REE prediction, underestimating it by − 5.6% as compared to IC [34]. In healthy Chilean individuals of both sexes, the Oxford equation also seems to be the best alternative for calculation of REE [39].

In our study, most predictive equations underestimated REE when compared to the reference criteria (− 9.1 to − 2.4% difference). In addition, we found a wide difference between measured and estimated REE, since the equations cannot estimate values with the same consistency and magnitude as IC. Similar discrepancies were also observed in other studies of patients with type 2 diabetes [33, 34].

Sex is a factor that has been associated with REE because men and women have differences in body size and composition [26–28]. When comparing the FAO/WHO/UNO equation in men and women, we found that it underestimated REE in both (− 1.6% vs. -1.8%, respectively). Conversely, in a study of French patients with type 2 diabetes, this equation overestimated REE in both sexes [29]. In another study of Brazilian women with type 2 diabetes, the equation also overestimated REE when compared to IC [33].

The Harris-Benedict equation is that most used in clinical practice to determine energy requirements [4]. However, studies have shown that it may not be appropriate to estimate REE in both sexes [40, 41]. In men and women without diabetes, the equation overestimated REE by 9% [40] and 14% [41], respectively. In our sample of individuals with diabetes, however, this equation underestimated REE in both men and women (− 1.9% vs. -3.1%, respectively). These findings are consistent with those of other studies which evaluated the accuracy of this equation in patients with type 2 diabetes [10, 30, 34].

The American Dietetic Association (now the American Academy of Nutrition and Dietetics) previously recommended use of the Mifflin-St. Jeor equation to estimate REE in overweight and obese individuals [42]. However, in our study, this equation was the one that most underestimated REE in men and women, with a difference of 152 kcal and 227 kcal/day, respectively. Similarly, the Schofield equation underestimated REE in both sexes (− 2.6% vs. -5.8%), while the Bernstein equation underestimated REE only in females (− 5.1%). These findings suggest that energy restriction calculations based on these equations may be insufficient to facilitate glycemic control and weight loss or maintenance in this population.

Most of the equations evaluated in this study were originally developed in healthy, eutrophic populations [4, 6–8, 10]. Thus, the differences we observed may have been due to the presence of obese patients (BMI > 30 kg/m^2^) in our sample, as well as to the fact that, in individuals with diabetes, insulin resistance is associated with abnormal metabolic reactions [43]. In fact, the presence of diabetes per se influences REE [9, 10, 14, 25, 32]. Studies conducted in Japan have shown that obese individuals with type 2 diabetes have a higher REE than their obese counterparts without type 2 diabetes, and that fasting blood glucose levels can be one of the main determinants of this increase [14, 25]. More recently, a study also performed in Japanese patients with type 2 diabetes showed that REE correlated significantly with plasma glucose and HbA1c [32]. The reasons for this phenomenon are not yet well established, but factors such as increased gluconeogenesis [9], increased protein turnover [44], increased glycosuria [9], and elevated levels of glucagon [45] may all influence REE in patients with diabetes. In fact, as already noted, studies have shown that the presence of diabetes is an important variable that must be considered when evaluating REE [9, 24, 25].

In 2002, Gougeon et al. evaluated the REE of women with type 2 diabetes and proposed an equation for predicting REE that included plasma glucose, HbA1c, and FM as independent variables [9]. In our study, however, although these variables were also considered we didn’t find significant correlations between REE and glycemic control in the group of our patients. This suggests that other metabolic factors, not controlled in our research, could influence REE in patients with type 2 diabetes. Moreover, we observed in this group of patients that the equation proposed by Gougeon et al. underestimated REE by 2.3% in both sexes. Other equations developed in patients with diabetes were also evaluated in our study. The equation by Huang et al. [10] overestimated REE with an 8.1% bias in both sexes. Martins et al. underestimated by − 7.5% in men and − 5.6% in women [11]. Different results were found in a study with Brazilian women with type 2 diabetes, in which the Gougeon equation overestimated REE by 2.8% and Hugan et al. equation underestimated by 11.2% [33].

The results of our study indicate that the DRIs equations to predict REE do not have an acceptable level of precision when applied to Brazilian patients with type 2 diabetes. In our study, these equations estimated higher REE values ​​when compared to the values ​​measured by IC, overestimating in men and women by 14.0 and 7.8% respectively. In a recent study carried out with the elderly, this equation had a bias of − 7.2% in men and − 6.6% in women [46]. Other study she was reported as accurate to estimate REE in men and women [47, 48].

The mean REE in the sample as a whole, measured objectively by IC, was 1644.6 ± 310 kcal/day. We found that men with type 2 diabetes had a higher REE (≅ 324 kcal/day) when compared to women. This corroborates previous studies conducted in obese individuals, which also demonstrated a higher REE in men [26–28]. It is well established that body composition differs significantly between men and women [49], and the variability in REE found between the sexes is probably because men have greater overall body mass and FFM than women. In our sample, we found significant correlations (*p* < 0.001) of REE with FM and FFM. REE correlated, albeit weakly, with FM in men (0.482) and with FFM in women (0.492). Studies have shown that including body composition (FM and/or FFM) in REE predictive equations does not improve their accuracy [31]. This is a relevant finding, because equations based on anthropometric parameters (weight and height) are more viable in clinical practice than equations based on body composition.

Our study had some limitations. Seasonality may influence REE, and our protocol was carried out over a 1-year period, thus including all seasons. This may have influenced the REE, as the climate is one of the factors that influences its variability. In fact, some studies have already shown a higher REE in winter than in summer [50, 51]. However, to minimize these effects we standardized the temperature and humidity of the environment where IC was performed so as to mitigate any seasonal influence on REE. The use of antidiabetic agents, antihypertensive agents and lipid-lowering agents by patients may have been a limitation, as these medications are known to induce metabolic alterations in individuals with type 2 diabetes. This effect was minimized by instructing the patients to take their first dose of the day only after REE measurement had been performed. On the other hand, this is the first study performed in Brazilian patients with type 2 diabetes to include sex stratification.

## Conclusions

Our findings suggest there is wide variability in the accuracy of predictive equations for REE. In addition, among the selected prediction equations, the BMR estimated by Oxford (≅ 54 kcal / day) and FAO / WHO / UNO (≅ 65.6 kcal / day) showed the smallest differences for men and women, respectively. The both equations, use weight and age at different cohort points, presented the best results when compared to IC. One explanation for this may be that these equations, derived from an ethnic population similar to that of Brazil, are based on a population mainly of European descendants. For clinical practice, the accuracy of the REE equations should be as appropriate as possible to promote the effectiveness of dietary advice and treatment of diabetes. In this sense, in the absence of IC, we recommend that in Brazilian patients with type 2 diabetes, the Oxford equation (for men) and the FAO / WHO / UNO equation (for women) are the best options for estimating REE.

## Supplementary information

**Additional file 1: Supplement 1.** Selected equations for estimating resting energy expenditure (REE).

## Data Availability

Data from this study are available upon reasonable request to the corresponding author.

## Abbreviations

DM: Diabetes mellitus
TEE: Total energy expenditure
REE: Resting energy expenditure
IC: Indirect calorimetry
VO_2_: Oxygen consumption
VCO_2_: Carbon dioxide production
