# Supplementary material for: Predictive equations for evaluation for resting energy expenditure in Brazilian patients with type 2 diabetes: what can we use?
Source: BMC Nutr. 2020 Sep 30;6:56. doi: 10.1186/s40795-020-00384-1 (PMC7525981; doi:10.1186/s40795-020-00384-1)
Supplement: Supplementary file 1 — Additional file 1: Supplement 1. Selected equations for estimating resting energy expenditure (REE). [file 40795_2020_384_MOESM1_ESM.docx]

**Supplement 1.** Selected equations for estimating resting energy expenditure (REE)

| **Reference** |  | | **Predictive equation** | **Number of subjects** | **Sample characteristics** |
| --- | --- | --- | --- | --- | --- |
| **Harris-Benedict** [4]  Men  Women |  | | 66 + [13.8 x W (kg)] + [5.0 x Ht (cm)] – [6.8 x (A)]  655 [9.5 x W (kg)] + [1.9 x Ht (cm)] – [4.7 x (A)] | 136 men  103 women  94 children | Healthy |
| **Bernstein** [5]  Men  Women |  | | (11.0 x W) + (10.2 x Ht) – (5.8 x A) – 1032  (7.48 x W) + (0.42 x Ht) – (3.0 x A) + 844 | 48 men  154 women | Obese |
| **Schofield** [6]  Men, age < 60 years  Men, age > 60 years  Women, age < 60 years  Women, age > 60years |  | | [0.048 x W (kg) + 3.653] x 239  [0.049 x W (kg) + 2.459] x 239  [0.034 x W (kg) + 3.538] x 239  [0.028 x W (kg) + 2.755] x 239 | 7173  men/women | Healthy adults |
| **FAO/WHO/UNO** [7]  Men, age < 60 years  Men, age > 60 years  Women, age < 60 years  Women, age > 60 years |  | | 11.6 x W (kg) + 879  13.5 x W (kg) + 487  8.7 x W (kg) + 829  10.5 x W (kg) + 596 | 11000  men/women | Healthy adults |
| **Mifflin–St. Jeor** [8]  Men  Women |  | | [W (kg) x 10] + [Ht (cm) x 6.25] – [(A) x 5] + 5  [W (kg) x 10] + [Ht (cm) x 6.25] – [(A) x 5] + 5 -166 | 251 men  247 women | Healthy adults |
| **Gougeon et al.** [9]  Men and women |  | | 375 + (85 × W) – (48 × FM) + (63 × FPG) | 25 men  40 women | Patients with type 2 diabetes |
| **Huang et al.** [10]  Men and women |  | | 71.767-(2.337xA)+(257.293Xsex)+(9.996xWt)+(4.132xht)+(145.959xDSI) | 1088  men/women | Obese with and without diabetes |
| **Martin et al.** [11]  Men  Women |  | | 909.4+(0.3505×A)×(BMI−34.524)-(135xRace)+(15.866xFM)-(9.10xDSI)  803.8+(0.3505×A)×(BMI−34.524)-(135×Race)+(15.866×FM)+(50.90×DSI) | 166  men/women | Lean an obese without diabetes |
| **Dietare Referece Intakes** [12]  Men  Women  Men  Women | |  | BMI 18.5 < 25 kg/m² :  662 - (9.53 x A) x (15.91 x W [kg] + (539.6 x Ht [m])  354 – (6.91 x A) x (9.36 x W [kg] + (726 x Ht [m])  BMI > 25 kg/m² :  293 – (3.8 x A) + (456.5 x Ht [m]) + (10.12 x W [Kg])  247 – (2.67 x A) + (401.5 x Ht [m]) + (8.6 x W [Kg]) | men/women | Normal-weight, overweight and Obese |
| **Oxford** [13]  Men, age 30–60 years  Men, age > 60 years  Women, age 30–60 years  Women, age > 60 years |  | | 14.2 x weight + 593  13.5 x weight + 514  9.74 x weight + 694  10.1 x weight + 569 | 800 men  5000 women | Healthy adults |
| **Ikeda et al.** [14]  Men and women |  | | (10 × Wt) − (3 × A) + (125 × Sex) + 750 | 68  men/women | Patients with type 1 and type 2 diabetes, lean and overweight |

BMI, body mass index; W, weight; A, age; Ht, height; FM, fat mass; FPG, fasting plasma glucose (mM); Sex: 1 for men and 0 for women; Race: 1 for black and 0 for white; DSI: 1 for patient with diabetes and 0 for healthy; PA, physical activity.
